# An Androgenic Agricultural Contaminant Impairs Female Reproductive Behaviour in a Freshwater Fish

**DOI:** 10.1371/journal.pone.0062782

**Published:** 2013-05-03

**Authors:** Minna Saaristo, Patrick Tomkins, Mayumi Allinson, Graeme Allinson, Bob B. M. Wong

**Affiliations:** 1 School of Biological Sciences, Monash University, Melbourne, Victoria, Australia; 2 Centre for Aquatic Pollution Identification and Management (CAPIM), The University of Melbourne, Bio21 Institute, Melbourne, Victoria, Australia; 3 DPI Queenscliff, DPI, Melbourne, Victoria, Australia; University of Lethbridge, Canada

## Abstract

Endocrine disrupting chemicals (EDCs) are a large group of environmental pollutants that can interfere with the endocrine system function of organisms at very low levels. One compound of great concern is trenbolone, which is widely used as a growth promoter in the cattle industry in many parts of the world. The aim of this study was to test how short-term (21-day) exposure to an environmentally relevant concentration of 17β-trenbolone (measured concentration 6 ng/L) affects reproductive behaviour and fin morphology in the eastern mosquitofish (*Gambusia holbrooki*). The mosquitofish is a sexually dimorphic livebearer with males inseminating females using their modified anal fin, the gonopodium, as an intromittent organ. Although the species has a coercive mating system, females are able to exert some control over the success of male mating attempts by selectively associating with, or avoiding, certain males over others. We found that females exposed to trenbolone approached males less and spent more time swimming away from males than non-exposed (control) females. By contrast, we found no difference in the behaviour of exposed and non-exposed males. Furthermore, exposure did not affect the anal fin morphology of males or females. This is the first study to demonstrate that exposure to an androgenic EDC can impair female (but not male) behaviour. Our study illustrates how anthropogenic contaminants can have sex-specific effects, and highlights the need to examine the behavioural responses of environmental contaminants in both sexes.

## Introduction

Over the last few decades, concern has been mounting over a group of environmental contaminants known as endocrine disrupting chemicals (EDCs). EDCs are causing concern because they disturb the endocrine function of organisms, often at very low concentrations (nanograms per litre levels), with potentially catastrophic effects. Infamous examples include eggshell thinning in birds [1], developmental abnormalities in alligators [2], and birth defects, gametogenesis and cervical cancer in humans [3], [4]. However, until now, studies have focussed mostly on estrogenic EDCs, with far less attention given to understanding the impacts of EDC pollutants with androgenic effects [5]–[9].

The androgenic steroid trenbolone acetate is widely used as a growth promoter in the beef industry in many parts of the world. In vivo, the compound is rapidly converted to the biologically active steroid 17β-trenbolone (hereafter referred to as trenbolone), which is an extremely stable compound, with a half-life up to 267 days measured in animal waste [10]. Trenbolone enters the environment through livestock urine and manure, and has been detected at levels from <5 ng/L to 20 ng/L in run off from cattle feedlots [11], [12] and up to 162 ng/L in fields receiving animal waste [13]. The morphological impacts of trenbolone on aquatic organisms, particularly fish, have been well documented, with effects ranging from reduced fecundity [6], [7] to complete sex reversal resulting in an all-male population [8], [9]. Our understanding of the behavioural effects of trenbolone exposure, however, is limited, even though behaviour has the potential to be a much more sensitive (and powerful) indicator of aquatic pollution than morphological biomarkers [14]–[19].

Trenbolone is known to bind to androgen receptors with three times the affinity of testosterone [20] and is therefore an extremely potent androgenic steroid in the environment. Considering its potency and the fact that androgens are known to affect the expression of sexual and agonistic behaviours, we would expect trenbolone to influence behaviour. So far, however, only two studies have specifically looked at the behavioural effects of trenbolone – and the results have been equivocal. Specifically, while embryonic exposure (50 ug) was observed to suppress copulatory behaviour in Japanese quail [21], trenbolone exposure (20 ng/L) had no effect on zebrafish courtship behaviour [8]. EDC-studies, to date, have also tended to focus only on male behaviour. In nature, however, both sexes are likely to be exposed to the same pollutants simultaneously and the effects on one sex could be very different in the other. As a result, it is important to investigate how EDCs might affect both males and females contemporaneously.

Recent studies have found that exposure to environmentally relevant concentrations of trenbolone can also induce morphological changes in fish. Ankley et al. [6], for example, found that female fathead minnows *Pimephales promelas*, exposed to trenbolone for 21 days developed dorsal tubercules – structures normally present on mature males. Also, trenbolone concentrations as low as 9.2 ng/L were found to cause irreversible masculinisation of zebrafish after 60 days of exposure [9]. Whether trenbolone exposure induces similar morphological changes in other species, however, remain unknown.

The eastern mosquitofish, *Gambusia holbrooki*, is an excellent model organism for studying the effects of androgenic EDCs because of its widespread, cosmopolitan distribution in shallow freshwater habitats in both urban and agricultural areas [22]. The mosquitofish is a sexually dimorphic livebearer, with males inseminating females using their gonopodium, as an intromittant organ [23]. Male mosquitofish do not court females but, instead, attempt forced copulations by thrusting their gonopodia towards the female’s genital pore [24], [25]. Despite the coercive mating system, evidence suggests that female mosquitofish are choosy [26]–[28] and may be able to exert some control over the success of male mating attempts by, for example, selectively approaching certain males over others [26]. Due to their internal mode of fertilisation, male mosquitofish need to be in close proximity to females before any mating attempts can be made, and both sexes clearly associate with each other during the breeding season [29]. Thus, as with other poeciliids [30]–[32], the time spent by females associating with males can have a direct bearing on mating outcomes and is a widely used measure of mating intentions in behavioural studies [28], [31], [33] Morphologically, previous research on mosquitofish has also found that embryonic exposure to androgenic hormones can increase the length of the modified anal fin (i.e. gonopodium) of males in relation to body size [34], [35], and induce gonopodial development in females [34], [36]–[37]. However, it is unknown whether EDCs might affect anal fin morphology once fish have reached maturity.

Accordingly, the aim of our study was to investigate the impact of trenbolone on male and female reproductive behaviour and fin morphology. In particular, we were interested in effects arising from short-term exposure to an environmentally relevant concentration (6 ng/L). This is ecologically important because agricultural pollutants enter the environment in pulses and previous work suggests that exposure to EDCs need not to be permanent to have long-lasting, detrimental effects [6]–[9], [38].

## Materials and Methods

### Ethical Statement

The methods for animal housing, handling and experimental protocols were assessed and approved by the Biological Sciences Animal Ethics Committee at Monash University (permit number: BSCI/2011/07). Because mosquitofish are a noxious species under State laws, the terms of the collecting permit (Department of Primary Industries Victoria, permit number NP191) did not allow them to be returned to the wild and hence fish were euthanised.

### Exposure Set up

Mosquitofish were collected from Brodies Lake in Victoria, Australia. This is a relatively pristine site located adjacent to a reservoir that supplies drinking water to parts of suburban Melbourne. Fish were caught during the breeding season (February) using dip nets and transported in coolers back to the laboratory. In total, 280 fish were collected, of which 140 were females and 140 males. Fish were separated by sex and acclimated to laboratory conditions (12∶12 h light regime) for 10 days in 54 L tanks (20 fish per tank). After acclimation, fish were randomly placed into separate-sex ‘exposure’ tanks (60 cm×30 cm×24 cm; 20 fish per tank), the set up of which followed the design of Saaristo et al. [17] with a few modifications. Briefly, 14 tanks were assigned to one of two treatments, namely, (1) a 17β-trenbolone exposed treatment (TB), and (2) a freshwater control. In total, 280 fish were exposed: seven tanks were allocated to the TB treatment (4 tanks for males and 3 tanks for females) and seven tanks were allocated to the control treatment (4 tanks for males and 3 tanks for females). We randomly took four fish from each of the holding tanks and placed them into each of the exposure tanks. This was continued until all of the fish from the holding tanks had been assigned to an exposure tank. Thus, each exposure tank had fish from several holding tanks. Male and female mosquitofish tanks in the TB treatment were exposed to trenbolone at a nominal concentration of 15 ng/L (measured concentration = 6 ng/L; see below for details on how trenbolone levels were monitored) via a flow-through system for 21-days. Mosquitofish tanks in the control treatment were connected to an identical, but separate, flow through system over the same period but, in contrast to the TB tanks, the flow through system supplied only freshwater to the fish during the exposure period. The water supplied to these fish tanks was fed through a mixing tank into which either trenbolone from a stock solution (in the case of the TB treatment) or freshwater (in the case of the control treatment) was pumped using a peristaltic pump (Watson Marlow 323 U/MC). From the mixing tanks, the water was channelled into the fish tanks using silicon tubing. The flow rate was kept constant (2.25 L/h) or all tanks using flow meters (BES Flowmeters, MPB Series 1200) and adjustable valves. For the trenbolone exposure, a fresh stock solution was prepared once a week and the stock solution tank was changed every third day to minimize the possible deterioration of TB. Water temperature in the tanks was monitored daily and ranged from 19–23°C. Fish were fed *ab litium* with commercial fish flakes (Otohime Hirame, Aquasonic) once a day during the exposure period.

### Monitoring of Trenbolone

The level of trenbolone used was achieved by firstly dissolving 17β-trenbolone (4,9,11-estratrien-17-ol-3-one; Novachem, Germany) in 100% ethanol (600 ug/L, 1% of ethanol) to create a stock solution, which was then diluted in the flow-through system to achieve the desired concentration. The final solvent concentration was 0.00006% in the exposure tanks.

The concentration of trenbolone in the exposure and control tanks was monitored by enzyme-linked immunosorbent assay (ELISA). To do this, a 100 mL water sample was taken from each exposure tank once a week. Water samples were acidified by adding a mixture of 1% acetic acid methanol, then loaded onto a conditioned solid phase cartridges (Strata×33 u, 500 mg,/6 mL; Phenomenex, Torrance, CA, USA). The cartridge was then eluted with methanol (2×4 mL), with the eluate dried under nitrogen stream. Samples were reconstituted with 100 uL methanol and 900 uL of deionised water.

Measurement of trenbolone was undertaken using commercial ELISA kits in accordance with the manufacturer’s instructions with a minor modification (Trenbolone ELISA kit; EuroProxima, Arnhem, The Netherlands). In short, a total of thirty samples and trenbolone calibration standards (freshly made in 10% methanol water) were dispensed (50 uL) in duplicate into an antibody coated 96 well plate by an auto dispenser (epMotion 5070, eppendorf, Hamburg, Germany). Thereafter, 25 uL of HRPO conjugate and 25 uL of antibody were dispensed into the wells. After 1 hour incubation at room temperature in the dark, the plate was washed three times with wash buffer by a microplate washer (Atlantis, ASYS HITECH, Eugendorf, Austria), and 100 uL of substrate was added to all wells. The plate was then incubated for a further 30 minutes at room temperature in the dark. Finally, 100 uL of stop solution was dispensed into all wells, and the absorbance of the solutions in the wells measured at 450 nm by a microplate reader (UVM40, ASYS HITECH, Eugendorf, Austria). Calculation of sample concentrations was undertaken by 4 parameter logistics method after creating a calibration curve using a series of standard calibration solutions (0, 0.125, 0.25, 0.5, 1.0, 5.0 ug/L) made up in 10% methanol. In order to verify calibration accuracy, check standards (i.e. standards from the kit run as samples) were run in duplicate on each ELISA plate during each ELISA test. The detection limit of trenbolone ELISA was 2.0 ng/L. The ratio of nominal concentrations and measured values were 90%, which indicates that the calibration curve provided good (accurate and precise) sample concentration values provided the ELISA response was within the upper and lower bounds of the calibration curve. A spike recovery experiment was conducted in triplicate using a 5 ng/L 17β-trenbolone solution. The average recovery was 97%, providing confidence that trenbolone in water samples was efficiently extracted, and that measured values were neither under nor over estimates of sample concentrations.

### Behavioural Trials

All behavioural trials were conducted in tanks (60 cm×30 cm×24 cm) containing freshwater with a 2 cm layer of gravel on the bottom as substrate. One male and one female from the same treatment group (i.e. either TB or control) were randomly assigned to an experimental tank and allowed to freely interact. We specifically paired fish from the same treatment groups because, in the wild, both sexes would typically be exposed to the same environmental contaminants simultaneously. Male and female behaviours were recorded with a video camera. Filming began when the male and female were released into the tank. Fish were filmed for 15 minutes and the behaviour of each sex was analyzed using JWatcher software, which calculates the total time and frequency of each quantified behaviour during this period. For females, we quantified whether or not the female was interacting with the male and, if so, whether she was actively associating with the male (i.e. swimming towards him), exhibiting aggressive behaviour (i.e. biting and performing tale beats), or trying to avoid the male by swimming away from him. For males, we quantified whether or not the male was showing an interest in the female. If so, we noted whether the male was orienting towards the female (within 5 cm of her body), chasing her, or engaging in gonopodial thrusts. We also noted the time the male took to perform the first chase. Trials were replicated 19 times for the trenbolone treatment and 18 times for the control. Each trial had a new pair of fish. We used 12 tanks for behavioural trials and ran 12 trials per day (6×TB and 6×control). For each tank, we alternated between TB and control trials to avoid tank effects.

### Morphological Measurements

After each behavioural trial, fish were euthanized with an overdose (40 mg/L) of anaesthetic clove oil [39]. Fish were then weighted and measured from the tip of the snout to the end of the caudal fin, and preserved in 70% ethanol for further anal fin measurements. The male gonopodium and female anal fin were analysed using the morphometric analysis described by Angus et al. [34]. The anal fin was photographed using a moticam 3.0 mounted on a Motic SMZ-168 stereomicroscope. From these images, ray 4 and ray 6 were measured to the nearest 0.001 mm using Motic Digilab II (Motic Instruments Inc., Hong Kong). The R4:R6 ratio is known to be influenced by EDCs in both the male gonopodium [40], [41] and the female anal fin [35], and was thus calculated (dividing length of R4 by R6) for both sexes.

### Statistical Treatment of Data

Data was checked for normality and heterogeneity of variance. In our analyses of female and male behaviour, the data did not conform to a normal distribution and we were unable to render them normal with transformation. Therefore, the effect of treatment on behavioural variables were analysed using Mann-Whitney tests. For the morphological (length and anal fin) data, independent-sample t-tests were used to test differences between TB-exposed and control fish. SE = standard error of mean. All statistical analyses were performed using SPSS (19.0).

## Results

### Female Behaviour

**Figure 1:**
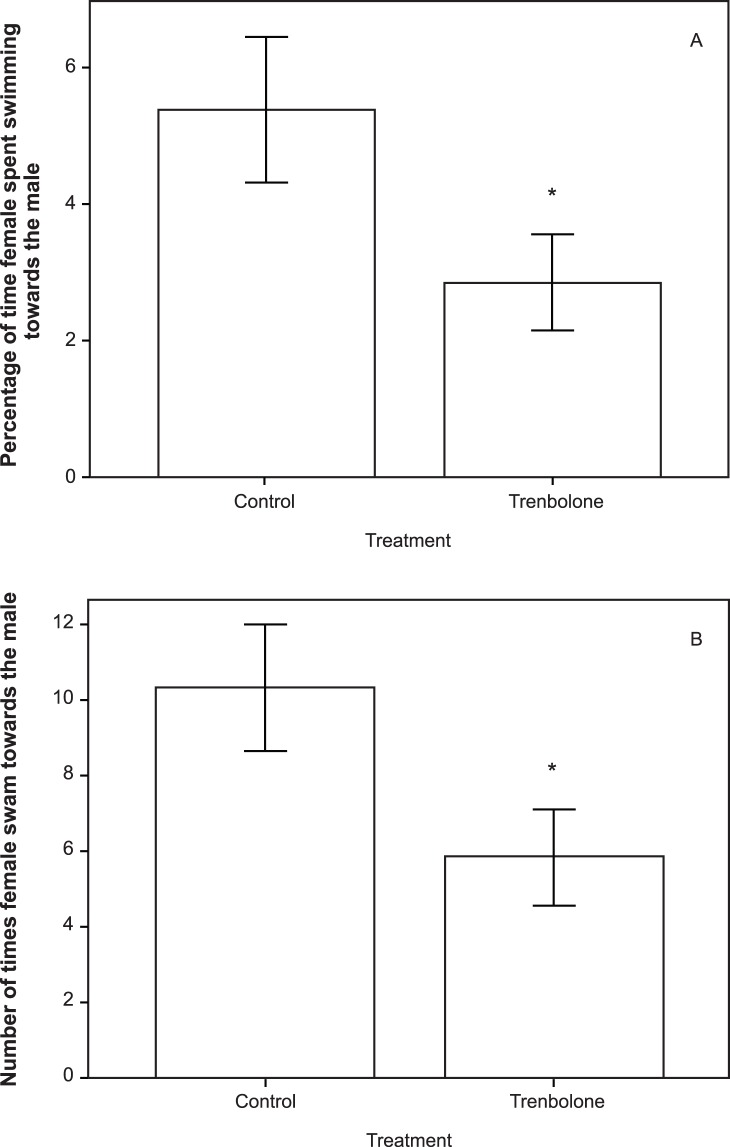
Mean percentage (± SE) of (A) time and (B) frequency females spent swimming towards the male during each trial. The two treatments are: Control = fish exposed to freshwater (n = 18), and Trenbolone = fish exposed to 6 ng/L of 17β-trenbolone (n = 19); Asterisk indicates a significant difference (p<0.05) between the TB treatment and control.

**Figure 2:**
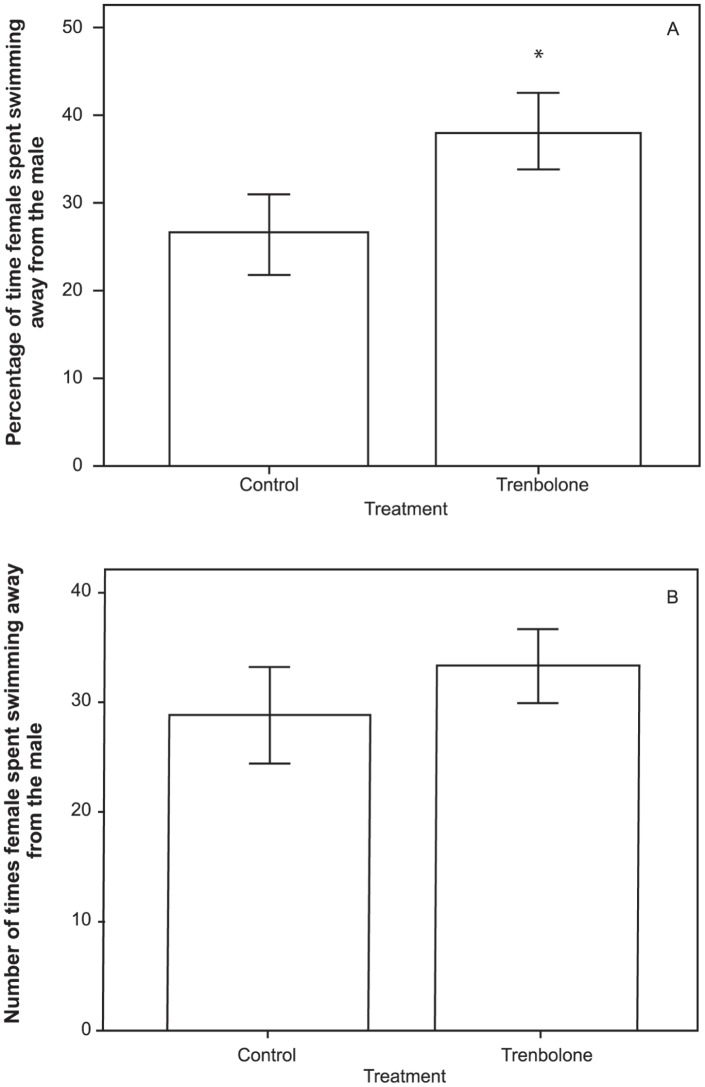
Mean percentage (± SE) of (A) time and (B) frequency females spent swimming away from the male during each trial. The two treatments are: Control = fish exposed to freshwater (n = 18), and Trenbolone = fish exposed to 6 ng/L of 17β-trenbolone (n = 19); Asterisk indicates a significant difference (p<0.05) between the TB treatment and control.

Trenbolone-exposed females spent less time associating with the males (duration of time (ms): Mann-Whitney: U = 106.000, p = 0.048, n = 37; number of times: U = 102.000, p = 0.035, n = 37; Fig. 1a,b). Females, instead, spent more time swimming away from the males (duration of time (ms): Mann-Whitney: U = 102.000, p = 0.036, n = 37; Fig. 2a), although the frequency of this behaviour did not differ between the treatments (Mann-Whitney: U = 127.000, p = 0.181, n = 37; Fig. 2b). Aggressive behaviours were not significantly affected (all p>0.05).

### Male Behaviour

Trenbolone-exposed males did not differ in behaviour from control males (Table 1). Males chased females in all of the trials and there was no difference in the time they took to perform the first chase between treatments (Mann-Whitney: U = 146.000, p = 0.417, n = 37; Mean ± SE: Control = 1.37 min ±0.454; TB = 1.67 min ±0.450).

**Table 1:** Effect of treatment on male behaviours.

	Mean ± S.E.		Test	
			Two sample t-test	
*Length (mm)*	Trenbolone	Control	t	p
Male	26.28±0.812	26.53±0.498	−0.264	0.793
Female	36.44±1.874	32.74±1.363	0.116	1.613
			Mann-Whitney test	
*Male behaviours*	Trenbolone	Control	U	p
Proportion of time				
Orientation	0.630±0.050	0.524±0.078	150.0	0.523
Chasing	0.016±0.002	0.015±0.003	149.0	0.504
Gonopodium thrust	0.004±0.001	0.003±0.001	144.0	0.412
No display	0.345±0.050	0.456±0.080	152.0	0.564
Frequency				
Orientation	46.67±4.506	43.16±5.962	149.0	0.504
Chasing	38.72±4.417	34.11±6.139	135.5	0.280
Gonopodium thrust	14.72±2.266	13.21±2.412	148.5	0.493
No display	9.89±1.188	11.89±1.644	144.0	0.411

The two treatments are: Control = fish exposed to freshwater (n = 18), and Trenbolone = fish exposed to 6 ng/L of 17β-trenbolone (n = 19). S.E. = standard error of mean.

### Morphological Analysis

The standard length of females and males (mean ± SE) did not differ between treatments (Females: two sample t-test: t = −1.367, df = 38, p = 0.180; Mean ± SE: control = 32.65 mm ±1.296, TB = 35.65 mm ±1.772; Males: t = −0.264, df = 38, p = 0.793; Mean ± SE: control = 26.53 mm ±0.498, TB = 26.28 mm ±0.812). Moreover, the R4:R6 fin ray length ratio did not differ between treatments for either male or female fish (two sample t-test: Males: t = 0.217, df = 38, p = 0.828, Mean ± SE: 2.28±0.055; Females: t = 0.208, df = 38, p = 0.701, Mean ± SE: 1.15±0.017).

### Trenbolone Measurements

The concentration of TB in the exposure tanks was 6 ng/L (SE = 2.6, n = 21). The concentration of TB in the control tanks was below detection limit throughout the exposure period. Details regarding nominal and actual water concentration of trenbolone are presented in Table S1.

## Discussion

We found that female and male mosquitofish responded differently to trenbolone. Trenbolone-exposed females approached males less and spent more time swimming away from males. This was true even though exposed males did not differ in their behaviour from control males. Because short-term exposure affected only female behaviour, this finding suggests that females may be more sensitive to trenbolone than males. To our knowledge, this is the first study to not only show that exposure to an environmentally relevant concentration of androgenic EDC can impair female reproductive behaviour, but that the behavioural consequences of EDC exposure can differ between the sexes.

When females were exposed to trenbolone, they approached males less often. A previous study by Toft et al. [42] showed that lifetime exposure to androgenic paper mill effluent decreased the time mosquitofish females stayed close to the male. The authors of that study described this as social ‘attending’ behaviour, but in the light of our findings, females might also have actively avoided the close distance of males. Although mosquitofish breed via a coercive mating system, females can nevertheless exert some control over fertilisation success and skew copulations by actively approaching and associating with certain males over others [26]. Hence, exposure to trenbolone could have implications for females by affecting their motivation to mate. Lack of interest is supported by the fact that exposed females actually spent more time swimming away from males even though trenbolone males did not harass females more. The impact of trenbolone on male mating behaviours, however, was less clear.

Testosterone is known to mediate male aggression and courtship behaviour [43]. Therefore, we hypothesised that trenbolone, which is more potent than testosterone, would increase male harassment (e.g. chasing, or engaging in gonopodial thrusts) of females. However, we did not find this to be the case. Why? Androgens, such as testosterone, are typically converted to estrogens in target tissues [44]. This increased concentration of estrogenic hormones can lead to a down-regulation of androgen production [43], which can in turn influence behaviour. However, this scenario is unlikely to occur with trenbolone. Previous work has shown that trenbolone is relatively non-estrogenic, because it is not a substrate for the aromatase enzyme that converts androgens to estrogens [20]. Therefore, exposure to trenbolone is likely to impact behaviours that are controlled by androgens rather than estrogens. It is unknown what impact trenbolone might have at higher concentrations on males but in our study, 6 ng/L was not sufficient to induce any significant behavioural changes. Clearly, further investigation into the behavioural impacts of trenbolone is required.

We did not observe any abnormal anal fin development amongst trenbolone-exposed males or females. Hormonally-dependent processes, such as anal fin development, are known to be particularly sensitive to EDC exposure [34], [36]–[37], [40], [45]–[46] Recent research has revealed that trenbolone can also influence gonopodial development in mosquitofish, with Brockmeier et al. [47] and Sone et al. [48] observing masculinisation of the female anal fin amongst trenbolone-exposed mosquitofish after 21 and 28 days of exposure respectively. However, in contrast to previously published studies, there are two possible reasons why we found no effect of trenbolone exposure on gonopodial morphology. First, we used a particularly low exposure concentration (6 ng/L compared to 10 µg/L used by Brockmeier et al. [47] and Sone et al. [48]). Second, we only exposed adult fish to the hormone. The male gonopodium, which is under androgenic control [36], forms via elongation of the anal fin during sexual development [22]. Therefore future studies may benefit from exposing fish to trenbolone from birth through to sexual maturity.

What are the potential population-level consequences of trenbolone-induced changes to behaviour? Recent studies have suggested that altered behaviours could have important population effects [16]–[19], [49]–[53]. At the beginning of the breeding season, mosquitofish densities are typically low, and sex ratios are female-biased [54]. Moreover, despite the persistence of males in trying to secure matings, actual copulatory success is extremely low [55]. Thus, selective female association could play an important role in reproductive success, particularly at low densities. As we have shown, trenbolone-exposed females not only approached males less than control fish, but they also actively avoided them more. This suggests that at times of low population density, trenbolone exposure has the potential to impact reproductive success and overall population viability. Such a possibility warrants further investigation.

In conclusion, we showed that exposure to an environmentally relevant concentration of trenbolone affected female reproductive behaviour. During the last decade, research has demonstrated that estrogenic EDCs can weaken reproduction and reproductive behaviour in a wide range of species [18], [50]–[52], [56]–[57]. Androgens, such as trenbolone, however, have been neglected, even though laboratory and field studies have demonstrated severe morphological effects [5]–[7], [58]. Not only does our study uncover a previously unknown behavioural impact of exposure to androgenic EDCs, but highlights how anthropogenic contaminants can have sex-specific effects, thus underscoring the need to examine both female and male responses contemporaneously.

## Supporting Information

Table S1 **Trenbolone concentration in the control and exposure tanks. ELISA = enzyme-linked immuno sorbent assay. LOR = limit of reporting.**
(DOC)
