# Supplementary material for: An Androgenic Agricultural Contaminant Impairs Female Reproductive Behaviour in a Freshwater Fish
Source: PLoS One. 2013 May 3;8(5):e62782. doi: 10.1371/journal.pone.0062782 (PMC3643955; doi:10.1371/journal.pone.0062782)
Supplement: Table S1 — Trenbolone concentration in the control and exposure tanks. ELISA = enzyme-linked immuno sorbent assay. LOR = limit of reporting. (DOC) [file pone.0062782.s001.doc]

**Supplementary material**

Table S1. Trenbolone concentration in the control and exposure tanks. ELISA = enzyme-linked immuno sorbent assay. LOR = limit of reporting.

**
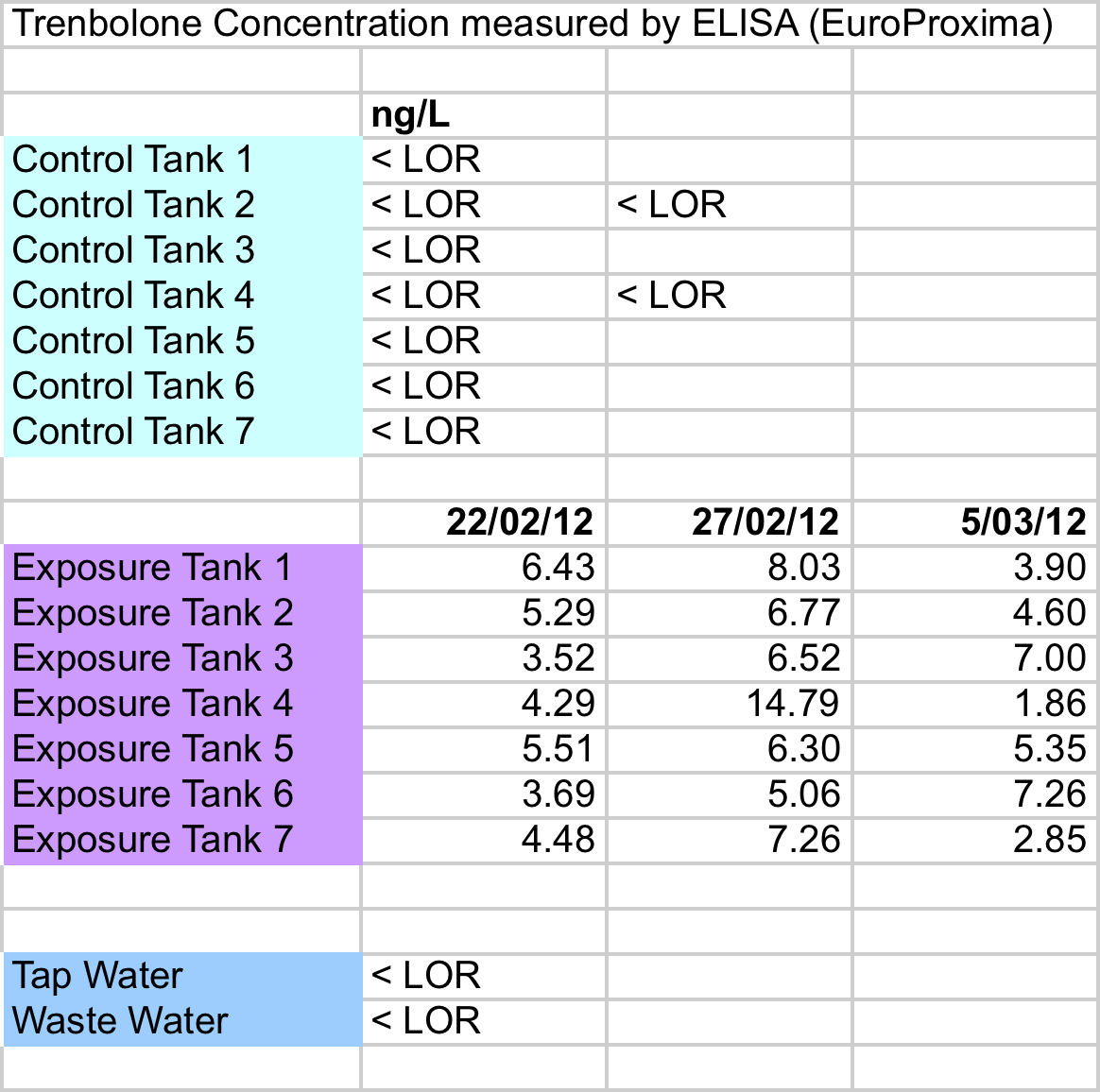
**
